# Enriched environment-induced neuroplasticity in ischemic stroke and its underlying mechanisms

**DOI:** 10.3389/fncel.2023.1210361

**Published:** 2023-07-07

**Authors:** Ping-Ping Han, Yu Han, Xin-Ya Shen, Zhen-Kun Gao, Xia Bi

**Affiliations:** ^1^Department of Rehabilitation Medicine, Shanghai University of Medicine and Health Sciences Affiliated Zhoupu Hospital, Shanghai, China; ^2^Department of Sport Rehabilitation, Shanghai University of Sport, Shanghai, China; ^3^Graduate School of Shanghai University of Traditional Chinese Medicine, Shanghai, China

**Keywords:** enriched environment, neuroplasticity, ischemic stroke, neurogenesis, angiogenesis

## Abstract

*Stroke* is a common cerebrovascular disease that can interrupt local blood flow in the brain, causing neuronal damage or even death, resulting in varying degrees of neurological dysfunction. *Neuroplasticity* is an important neurological function that helps neurons reorganize and regain function after injury. After cerebral ischemia, neuroplasticity changes are critical factors for restoring brain function. An enriched environment promotes increased neuroplasticity, thereby aiding stroke recovery. In this review, we discuss the positive effects of the enriched environment on neuroplasticity after cerebral ischemia, including synaptic plasticity, neurogenesis, and angiogenesis. In addition, we also introduce some studies on the clinical application of enriched environments in the rehabilitation of post-stroke patients, hoping that they can provide some inspiration for doctors and therapists looking for new approaches to stroke rehabilitation.

## Introduction

Ischemic stroke is a type of stroke that occurs when a blood vessel in the brain becomes blocked, and it is the most common type of stroke, accounting for approximately 87% of all strokes (Feske, 2021). It is a leading cause of death and disability worldwide. Cerebral ischemia leads to hypoxia and impaired energy metabolism, and intracellular redox homeostasis is disrupted, resulting in an enhanced oxidative stress response. Oxidative stress causes adverse reactions such as cell membrane lipid peroxidation, DNA damage, and mitochondrial dysfunction, exacerbating nerve cell damage. The impaired energy metabolism caused by cerebral ischemia results in cell death, of which apoptosis is an essential mode of cell death (Li et al., 2018). Apoptosis mainly proceeds through the mitochondrial and death receptor pathways, including molecular mechanisms such as cysteine protease initiation and cysteine aspartate protease activation, after brain ischemia, cell damage and necrosis release cytokines that activate immune cells and cause an inflammatory response (Zhang et al., 2022). The inflammatory response leads to pathophysiological changes such as brain tissue edema and blood-brain barrier disruption, exacerbating neuronal cell damage (Zhao et al., 2022). In addition, cerebral ischemia causes subcellular level damage, such as mitochondrial dysfunction, cytoplasmic enzyme activation, and nucleus DNA damage, leading to neuronal cell death (Kaur and Sharma, 2022). Although many patients have received standard rehabilitation during the acute phase, strokes can still result in long-term motor, speech, and cognitive impairment. This devastates the patient and places a heavy medical burden on the community. Therefore, seeking post-stroke rehabilitation methods to improve the prognosis is essential.

What is exciting is that the central nervous system is regenerative, called neuroplasticity, and this is the structural basis for functional recovery after a stroke. It has been widely demonstrated that an enriched environment can enhance neuroplasticity and, thus, functional recovery after ischemic stroke. This review will provide an overview of the effects of an enriched environment on neuroplasticity in ischemic stroke and its underlying mechanisms.

## Neuroplasticity in ischemic stroke

Neuroplasticity, also called brain plasticity, refers to the ability of the brain to change its structure and function in response to new environments or injuries. The concept of neuroplasticity can be traced back to the early 20th century. As early as 1890, neurologist Santiago Ramón y Cajal introduced the concept of “neuroplasticity.” By observing the morphological structure of neurons in the brain, he discovered that the connections between neurons are not fixed but can change based on experience and learning. In the second half of the 20th century, neuroplasticity was gradually studied in greater depth as neuroscience and technology advanced. One of the most important contributions was the “long-term potentiation” theory by Canadian neuroscientist Donald Hebb. He proposed that when neurons are activated simultaneously, the synaptic connections are strengthened, leading to learning and memory formation (Brown and Milner, 2003). Studies have shown that individuals who are experts in specific motor skills, such as musicians, typists, golfers, and basketball players, have specific structural differences in brain regions associated with the motor skills they have honed through extensive practice (Cannonieri et al., 2007; Park et al., 2009). For example, professional musicians tend to have higher gray matter density in the auditory, sensorimotor, and premotor cortices and cerebellum. Usually, the amount of practice correlates with structure in these areas. Similarly, professional typists have been found to have greater gray matter volume in the prefrontal cortex, supplementary motor area, and cerebellum, which correlates with typing experience. Expertise in golf and basketball has also been associated with structural differences in specific brain regions (Jäncke et al., 2009). These studies suggest that the brain adapts to the demands of specific skills, leading to structural changes in relevant brain areas.

After a stroke, neuroplasticity is a spontaneous process initiated to compensate for damaged cells and neural pathways, including axonal sprouting, neurogenesis, angiogenesis, etc. In addition, alterations in neural pathways and brain connectivity can be found in the lesion’s periphery, and such alterations can be detected even in the interhemispheres (Dimyan and Cohen, 2011; Small et al., 2013). In ischemic stroke, the infarct core refers to the necrotic area of brain tissue due to ischemia and hypoxia. The area around the infarct core is called the penumbra, the peri-infarct cortex. It is the area of brain tissue affected by ischemia and hypoxia that has reduced blood flow but still has a residual blood supply (Kearns et al., 2022). Penumbra may gradually regain function after stroke because their neurons remain damaged and not completely dead. *In vivo* two-photon imaging found that stroke causes damage to dendrites within the penumbra, but some damaged structures can be restored during reperfusion. It is important to note, however, that the process of neuronal survival in the penumbra is time-limited, and without timely intervention, cells may die within hours or days (Dirnagl et al., 1999). After a stroke, the mechanisms of neuroplasticity are crucial for restoring and reconstructing neurological functions, including the reconstruction and regeneration of neuronal synapses and compensatory neuronal proliferation after neuronal death. Recovery after a stroke is not a recovery of the original nerve cells and pathways but a compensatory repair, that is, the replacement of the damaged cortical functions by other cortical parts of the brain. Cortical re-projection can occur in the damaged area to facilitate recovery (Carmichael, 2003). This reprojection is a neuroplasticity manifestation, allowing undamaged neurons to receive signals from the damaged area. Such re-projection usually happens in the penumbra, which can take over the function of the damaged area and replace the lost function by reconnection (Liu et al., 2008; Stokowska et al., 2017). In addition, neurons in the peri-infarct region can be reorganized and reconstructed by establishing new synaptic connections and growing synapses (Joy and Carmichael, 2021; Yamagata, 2021). Although spontaneous remodeling changes occur in the brain of patients with cerebral ischemia, the effects of these changes are minor and cannot bring about significant functional improvement (Tennant et al., 2017). Therefore, it is necessary to seek rehabilitation methods that promote neuroplasticity actively.

## Enriched environment-induced neuroplasticity in stroke recovery

### What is an enriched environment?

**FIGURE 1:**
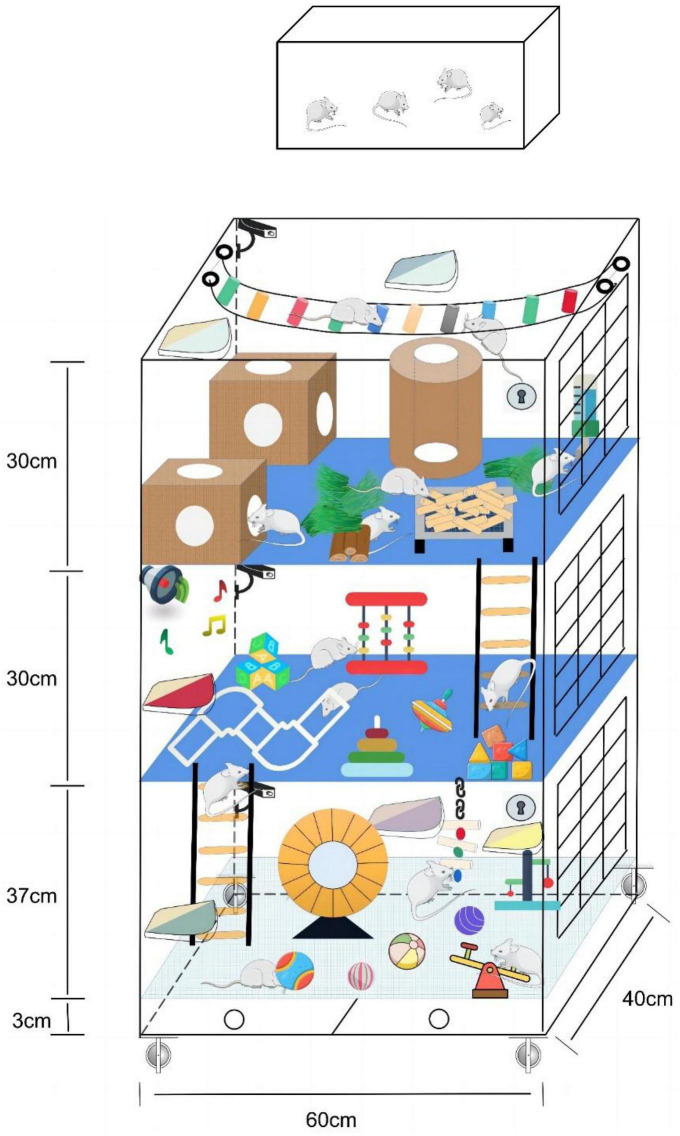
Comparison chart of standard and enriched environments. The top is the standard environment, and the bottom is the enriched environment.

Mark Rosenzweig, a prominent figure in environmental enrichment, described enriched enrichment as a combination of inanimate and social stimulation (Rosenzweig and Bennett, 1996). In an enriched environment, animals are exposed to sensory, motor, and social experiences that can significantly influence brain function and behavior. Studies using enriched environments have demonstrated that they can enhance learning, memory, and brain plasticity and even offer protection against neurological disorders like Alzheimer’s disease (Balthazar et al., 2018; Hua and Min, 2020; Li et al., 2021; Stazi et al., 2023). As a result, enriched environments have become a vital tool in neuroscience research, helping researchers understand the mechanisms of brain plasticity and develop interventions to improve brain function. Figure 1 is a schematic diagram of the standard and enriched environment. This enriched environment cage was designed and patented by our group. In the last year, we have used this cage to conduct studies related to enriched environments and ischemic stroke. The corresponding article is currently being written.

In animal research, an enriched environment refers to a more stimulating and challenging environment for animals that differs from standard laboratory animal housing conditions. Such environments often include larger cages or exercise areas, great toys and equipment, diverse food and water sources, more social interactions and activities, and more opportunities for exploration and learning (Varty et al., 2000; Zentall, 2021). Enriched environments positively affect animals’ physiological and behavioral states, such as increasing the number of neurites and synapses in the brain, increasing neuroplasticity, improving memory and learning ability, and reducing stress anxiety. For example, enriched learning opportunities promote neurogenesis and maturation and increase neuronal connectivity in the brain, thereby improving memory and learning. Social interaction promotes the development of social cognition, and voluntary exercise promotes angiogenesis and neurotrophic factor production in the brain, thereby promoting neuronal growth and maturation (Pham et al., 2002; Kempermann, 2019). An enriched environment’s effects on the brain result from interactions among multiple essential elements. Although certain specific factors contribute to the importance of the enriched environment, such as socialization and spontaneous physical movement, a single variable does not fully achieve the enriched intervention effects of an enriched environment. This is because a single factor can only affect certain aspects of the brain and cannot encompass the complex effects of multiple factors in an enriched environment. Therefore, the brain can be fully stimulated and exercised only in an enriched environment, leading to broader cellular, behavioral, and cognitive changes (Ickes et al., 2000). Research on enriched environments has gradually expanded to the human domain in recent years. Some studies have shown that children in rich environments are likelier to achieve advantages such as good academic performance, improved attention, and attentional control than children living in monotonous environments. Moreover, among adults, those who receive stimulation from rich environments also show advantages such as greater cognitive flexibility and better retention of brain function. Enriched environments are often considered an actionable intervention. What is in the enriched environment is highly variable, and researchers can design different enriched environment paradigms according to the needs of their experiments (Modlinska et al., 2019). As a cornerstone of neuroscience research, the enriched environment paradigm is critical for studying brain plasticity and developing strategies to improve brain function in various contexts. Studies have shown that an enriched environment positively affects brain plasticity after stroke. A study in stroke rats found that placing stroke rats in an enriched environment promoted neurogenesis and recovery of synaptic plasticity, thereby increasing the rate and extent of recovery of motor and cognitive function. Another study found that neuronal regeneration and survival rates in damaged brain regions were higher for stroke rats living in an enriched environment than those living in a typical environment.

Enriched environments provide opportunities for increased sensory, cognitive, and motor stimulation and have been shown in animal studies to enhance post-injury recovery and brain plasticity. In a rich environment, animals are exposed to various sensory stimuli and complex tasks that encourage exploration, learning, and social interaction. These experiences have been shown to stimulate neurogenesis, synaptogenesis, and dendritic growth in various brain regions, including those involved in motor function. Studies have shown that post-stroke animals housed in enriched environments exhibit better functional recovery, including motor function and cognitive performance, than those housed in standard laboratory cages. Enriched environments have also been shown to enhance the effects of post-injury training, such as task-specific training, by promoting the formation of new neural connections and improving training effectiveness (Park et al., 2009). These findings suggest that combining post-injury training and an enriched environment may be a promising approach to enhance recovery after stroke and promote brain plasticity. However, it is worth noting that differences in the underlying biology and complexity of human stroke may limit the application of these findings to human stroke patients. Nonetheless, studies in animal models provide a valuable basis for developing more effective post-stroke rehabilitation strategies. Recovery from stroke depends on the ability to survive neural circuits to reorganize and compensate for damaged areas. Many studies have demonstrated the positive effect of the enriched environment on this process.

## Enriched environment and synaptic plasticity

The central issue in brain plasticity is the plasticity of synapses. Synapses are the connections between neurons that allow them to transmit information and communicate with each other (Cizeron et al., 2020). There are two main types of synapses: chemical synapses and electrical synapses. Chemical synapses are the most common type of synapse and transmit signals using neurotransmitters. A chemical synapse consists of a presynaptic membrane, a synaptic gap, and a postsynaptic membrane. The presynaptic membrane releases neurotransmitters, which travel across the synaptic gap to the postsynaptic membrane, triggering a change in potential and transmitting the information (Grant, 2019). Synaptic plasticity is a phenomenon in which the connections between neurons can change and adjust as the nervous system is stimulated and experiences new things. During synaptic plasticity, the neural information transmitted by synapses can be enhanced or diminished, thus changing the strength and function of the connections between neurons (Magee and Grienberger, 2020). Synaptic plasticity is a crucial component of neuroplasticity, a necessary means by which the nervous system adapts to the external environment and internal physiological changes. It is essential in various neural functions such as learning, memory, sensory processing, and neurodevelopment. The degree of damage to neuronal synaptic structures after cerebral ischemia is related to the duration and degree of infarction. Severe cerebral ischemia leads to irreversible neuronal damage and edema necrosis, whereas transient or mild to moderate cerebral ischemia is mainly reversible neuronal degeneration. Changes in synaptic ultrastructure occur differently after different degrees of cerebral ischemia. In transient cerebral ischemia, only a few dendritic spines are lost and can be recovered after improving adverse ischemic factors. In contrast, severe acute ischemia and hypoxia can lead to axonal and dendritic spine ablation, a sharp reduction in synapse number, and a widening of synaptic gaps in a short period.

Long-term potentiation (LTP) is a form of synaptic plasticity between neurons. It refers to the fact that when a synapse is repeatedly stimulated at high frequencies, its transmission efficiency can be enhanced for long periods, and this enhancement can last from hours to days (Malenka and Bear, 2004). LTP is one of the foundations of memory and learning in the brain. At neuronal excitatory synapses, high-frequency stimulation can lead to the massive entry of calcium ions, which bind to CaMKII (calcium/calmodulin-dependent kinase II) and activate CaMKII. CaMKII promotes the translocation of AMPA-type glutamate receptors (AMPAR) through phosphorylation, making them more susceptible to binding to glutamate in depolarized postsynaptic neurons. In addition, CaMKII can also promote postsynaptic signaling pathways such as MEK/ERK and CREB, which contribute to enhanced synaptic transmission and protein synthesis. LTP formation also involves other molecular mechanisms such as phosphorylation of target proteins, involvement of signaling pathways such as NO (nitric oxide), and protein synthesis in pre- and postsynaptic cells (Diering and Huganir, 2018). The interaction and coordination of these molecular mechanisms promote the formation and maintenance of LTP, thus enabling long-term synaptic plasticity and providing the molecular basis for learning and memory.

N-methyl-D-aspartate receptor (NMDAR) is an excitatory amino acid receptor, mainly located in the cerebral cortex, striatum, and hippocampus. They are coupled to ion channels to form receptor channel complexes. NMDAR has multiple subunits, including NR1h and NR(A-D), and these subunits can function in different combinations (Yang et al., 2012; Ge et al., 2020). Neuronal synaptic plasticity is closely related to NR1/NR2A and NR1/NR2B combinations. Reduced levels of NR2A phosphorylation and increased levels of NR2B phosphorylation can be observed in the late stages of reperfusion injury (Fan et al., 2014). A study on the effect of an enriched environment on cognitive function in rats with chronic cerebral ischemia found that an enriched environment upregulated the protein levels of NMDAR, NR2A, and NR2B in hippocampal and cortical regions as the expression of synaptic plasticity-related proteins and ultimately improved cognitive function. To further validate the important role of NMDAR in this process, the authors used MK801, a specific antagonist of NMDAR. They found that the positive effects brought about by the enriched environment were lost (Zhang et al., 2021b).

Post Synaptic Density (PSD) is a post-excitatory postsynaptic membrane super-signaling molecule complex, which is essential for synapses to perform their transmission functions. It is widely accepted that PSDs are situated on the postsynaptic membrane of dendritic spines in all chemical synapses within the central nervous system (Cook et al., 2012; Ugalde-Triviño and Díaz-Guerra, 2021). Morphological characteristics such as area, size, thickness, curvature, length, perforation status, and the size of perforations vary among synaptic connections in different neural pathways, neurons, and brain regions. Typically, a lack of density in the center of a perforation resembles the diameter of a hole. Synaptic perforation is believed to indicate a change in the functional state of the synapse or even an increase or decrease in the number of synapses. Moreover, PSD perforation could potentially enhance synaptic transmission by expanding the contact area of neurotransmitters (Huang et al., 2018). PSD-95 is the most abundant and vital scaffold protein and a member of the guanylate-related kinase family, mainly found in mature excitatory glutamatergic synapses. PSD-95 can bind to related receptors and signaling molecules through different structural domains to form signaling complexes, participate in synaptic junction formation and maintain synaptic plasticity (Mayor-Nunez et al., 2021). Synaptophysin (SYP, also known as p38) is a calcium-binding protein closely related to the structure and function of synapses. It is transported to the end of the axon after being synthesized in the neuron cell body and is distributed explicitly in the presynaptic vesicle membrane. Syp is a membrane protein closely related to the structure and function of synapses, and its quantity and distribution density can indirectly reflect the density of synapses. Its expression increases significantly during synaptic reconstruction, so it is often used as a molecular marker to highlight synaptic reconstruction. It was shown that Syp expression decreased in the early stages of cerebral ischemia, after which its expression gradually increased and then decreased to normal levels. By studying Syp expression in the CA1 region of the hippocampus of ischemic gerbils, Ishimaru et al. (2001) found that on day two after ischemia, Syp expression was significantly reduced in the hippocampus in the primordium, radial layer, and parietal dendrites of neurons in the CA1 region and disappeared in the white matter. However, at days 7 and 14 post-ischemia, deeply stained Syp immunoreactions were visible around surviving neurons in the CA1 region (Ishimaru et al., 2001). Ischemic brain injury stimulates the expression of cortical Syp around the ischemic focus, which promotes the budding of neural axons and the formation of new synapses, resulting in corresponding morphological changes in the ultrastructure of neural tissue. This morphological change serves as a functional basis to promote the recovery of neurological dysfunction after cerebral ischemia. Rönnbäck et al. (2005) used microarray techniques to demonstrate gene expression profiles in the hippocampus of rats with a stroke 1 month after environmental enrichment intervention, which showed high expression of the PSD-95 gene. One study’s results of western blots showed that SYN and PSD-95 expression levels were significantly higher in the ischemic hippocampal region 4 weeks after permanent middle cerebral artery occlusion in the enriched environment group. This group also had more synapses and better performance in the Morris water maze (Wang et al., 2019). Another study found increased synaptic density in the parietal cortex of mice undergoing MCAO-modeling in the intervention group, as well as structural changes in synaptic connections, reduced synaptic cleft width, and increased postsynaptic density (PSD) thickness in the parietal cortex and hippocampus, resulting in improved performance on spatial memory tasks. The investigators also found elevated expression of PSD-95 (Xu et al., 2009).

## Enriched environment and adult neurogenesis

Neurogenesis is a complex biological process involving the synergistic action of multiple stages and cell types. In the adult brain, the number of neurons is relatively constant, but neurogenesis makes the generation of newborn neurons in the adult brain possible (Cameron and Glover, 2015). Neurogenesis usually includes the following stages: the proliferation of neural stem cells (NSCs), differentiation of neural precursor cells (NPCs), and migration and maturation of newborn neurons. In the early stages of neurogenesis, neural stem cells continue to self-replicate, thus maintaining their number and function. Then, a portion of neural stem cells differentiate into neural precursor cells, and these cells further differentiate into new neurons or glial cells. The newborn neurons need to migrate to the area where they are going and make connections with the surrounding neurons (Yoo and Blackshaw, 2018). This process is called neuronal migration and is one of the most critical stages in neurogenesis. Neuronal migration involves a complex process of directional migration of neurons, formation and expansion of growth cones, and selection and association of target cells. Growth cones are protruding, elongated structures outside the neuronal cell body composed of cytoskeletal proteins such as microtubules and microfilaments. The formation and extension of growth cones is the main neuronal movement and migration mode. Growth cones are usually located at the ends of axons or dendrites of neurons and are formed by the polymerization of cytoskeletal proteins. During neural development, growth cones help neurons find the correct connection sites and establish connections with other neurons or target cells by relying on cytoplasmic dynamics to drive, extend and move them (Cameron and Glover, 2015). Finally, newborn neurons mature and begin to participate in the regulation of brain function. Many factors affect neural stem cell proliferation, including brain-derived neurotrophic factor (BDNF), insulin-like growth factor-1 (IGF-1), fibroblast growth factor-2 (FGF-2), and fibroblast growth factor-2 (FGF-2) (Kokaia et al., 1998; Yoshimura et al., 2001; Ikeda et al., 2005; Yan et al., 2006; Ruan et al., 2015). Also, previous studies identified factors influencing the migration process of neuroblasts: stroma-derived factor (SDF-1), monocyte chemotactic protein (MCP-1), and matrix metalloproteinase (MMP) 2,3 and 9 (Wang et al., 2006; Barkho et al., 2008).

Neurogenesis has become a topic of widespread interest since the concept of neurogenesis microenvironment was reported (Jin et al., 2003). The main sites of adult neurogenesis are located in the subventricular zone (SVZ) of the lateral ventricle, and the subgranular zone (SGZ) of the hippocampal dentate gyrus contains neural stem cells. Recent experimental evidence has demonstrated that neurological improvement after stroke may be induced by neuronal replacement of endogenous neural stem cell origin. Therefore, aiming to activate post-ischemic neurogenesis to replace dead neurons would be a promising area of research (Arvidsson et al., 2002).

The enriched environment has been shown to promote neurogenesis in various disease models, including anxiety, Down syndrome, Alzheimer’s disease, stroke, and more (Plane et al., 2008; Valero et al., 2011; Pons-Espinal et al., 2013; Ramírez-Rodríguez et al., 2022). The pathological process of cerebral ischemia causes the death of neural stem cells, but the exact mechanism remains unclear. Indeed, the location and degree of ischemia and hypoxia vary, as does the amount of NSCs death (Levison et al., 2001; Arvidsson et al., 2002). It has been reported that cerebral ischemia activates Notch signaling within neural stem cells, which initiates the conversion of NSCs from neurogenesis to gliosis. This conversion process is irreversible (Morrison et al., 2000; Pear and Simon, 2005). Several studies have found that the survival of newborn neurons decreases after cerebral ischemia (Seki and Arai, 1995). This is because of the lack of adequate trophic factors and inflammatory response in the microenvironment where these newborn neurons are located (Zhang et al., 2004; Salmeron et al., 2019). Cerebral ischemia leads to local hypoxia and inadequate nutrient supply, which can affect the supply of energy and nutrients needed during neurogenesis. The inflammatory response caused by ischemia interferes with signaling pathways during neurogenesis, which can affect neuronal survival and development (Salmeron et al., 2019). In addition, the rate of neurogenesis gradually decreases with age, which makes the elderly more susceptible to neurogenesis after cerebral ischemia.

Neurogenesis after cerebral ischemia occurs mainly in specific areas near the area of brain injury, such as the lateral subventricular zone and the hippocampus. These areas are rich in neural stem cells and precursor cells, which have a great potential to differentiate into many types of neurons and glial cells. After a stroke, the neurogenesis process is activated, promoting the repair and regeneration of neural tissue, a fundamental self-healing mechanism. Neurogenesis involves the regulation of multiple molecular, cellular, and signaling pathways, which include transcription factors, growth factors, extracellular matrix, neurotransmitters, neurotrophic factors, etc. Multiple factors, such as neural environment, neurotrophic factors, and immune response, influence neural stem cells’ localization, differentiation, and survival. Therefore, an in-depth understanding and control of the neurogenesis process are essential to promote the repair and regeneration of brain-damaged areas. Promoting post-stroke neurogenesis is a promising therapeutic strategy to help patients restore brain function and improve their quality of life.

It is encouraging to note that the enriched environment also has a non-negligible role in promoting post-stroke neurogenesis. A study by Matsumori et al. (2006) found that after the same middle cerebral artery occlusion, there was more proliferation of neural progenitor cells and an increase in neuroblasts within the hippocampal dentate gyrus of rats residing in enriched environment cages, although this rise was transient. The study also found that the number of NeuN-positive cells in the ischemic penumbra was more significant in the enriched environment group (Matsumori et al., 2006). Another study on the enriched environment and experimental stroke found similar results, with proliferation and differentiation of neural stem cells being more pronounced in the enriched environment group and the rats in this group also achieving better performance in the rotarod test (Komitova et al., 2005). During development, retinoic acid (RA) plays a vital role in regulating neurogenesis, and RA continues to be expressed in the adult brain. It has been found that the differentiation of dentate granule cells is also reduced in adult rats lacking RA (Maden, 2002, 2007; Jacobs et al., 2006). Thus Jennifer M. Plane and her colleagues examined the effects of retinoic acid (RA) combined with an enriched environment on neurogenesis in post-stroke rats. MRI (Magnetic Resonance Imaging) and immunofluorescence results showed that RA+ enriched environment treatment preserved striatal and hemispheric tissue and promoted neurogenesis in the SVZ and striatum (Plane et al., 2008). These results suggest the possibility that pharmacological treatment combined with an enriched environment contributes to neurogenesis and functional outcome after stroke. Studies on the enriched environment treatment after post-stroke neurogenesis are still scarce, and the mechanisms behind them are still unclear. However, existing studies have shown the existence of post-ischemic neurogenesis and its importance for functional recovery after stroke, so more in-depth researches are necessary.

## Enriched environment and angiogenesis

Angiogenesis, the process by which blood vessels in the body can grow and repair again, is essential for recovery after a stroke. It occurs around the damaged area, mainly by the migration of precursor cells such as endothelial progenitor cells and bone marrow stem cells in the brain to the damaged area, differentiates into endothelial cells and smooth muscle cells, and proliferates to form new Blood vessels (Hatakeyama et al., 2020; Fang et al., 2023). Angiogenesis and neurogenesis are simultaneous processes after stroke, with close interaction and influence between them (Ruan et al., 2015). Both work synergistically to promote the restoration of damaged brain tissue and function. Angiogenesis can provide the necessary blood flow and nutrients for neurogenesis and produce molecules such as vascular endothelial growth factor (VEGF), which promote neurogenesis and connection formation. At the same time, neurons can promote angiogenesis and recovery by secreting molecules such as nerve growth factors (Paro et al., 2022). An animal experiment found that mice in the enriched environment group had higher microvessel density (MVD) in the ischemic border zone compared to the standard environment group; those growth factors that promote blood vessel formation and maturation, such as vascular endothelial growth factor (VEGF) and angiopoietin -1 and its receptor (Ang-1/Tie-2) expression levels were also increased (Zhang et al., 2021a). In addition to the peri-infarct area, experiments have shown that an enriched environment can increase the number of blood vessels in the cingulum and its periphery (Shen et al., 2020). The signaling pathways may include the astrocytic high-mobility group box-1/interleukin-6 (HMGB1/IL-6) and PI3K/AKT/GSK-3/β-catenin signaling pathway (Chen et al., 2017; Zhan et al., 2020). Similar results were found in another experiment, in which an enriched environment intervention increased the total vessel surface area in the peri-infarct area. In addition, the number of branch points also increased (Zhang et al., 2017). Table 1 summarizes animal studies on the enriched environment enhancing neurological plasticity after cerebral ischemia.

**TABLE 1:** Enriched environment improves post-stroke neuroplasticity and its underly mechanisms in animal studies.

Ischemia model	Housing condition	Time of intervention	Outcomes	Mechanisms	References
pMCAO	The cage was 65 cm wide, 75 cm long, and 25 cm high, with climbing ladders, plastic tubes and tunnels, running wheels, and small boxes. For environmental novelty, things were changed every 3 days.	Starting after 3 days of pMCAO and lasting for 25 days	The results indicate that an enriched environment positively mitigates the spatial learning and memory deficits caused by permanent middle cerebral artery occlusion.	Enriched environments increase the number of hippocampus synapses and synaptic plasticity by upregulating GAP-43, SYN, and PSD-95.	Wang et al., 2019
tMCAO	Toys, wooden blocks, running wheels, Plexiglas tunnels, ladders, plastic castles, swings, and more were in the 2 m × 2 m × 1 m cage. Rats in an enriched environment spent 10 min in a 120 cm-diameter open field during cage re-decoration. Enriched environment housing and field objects were refreshed daily.	Starting after 3 days of pMCAO and lasting for 14 days	An enriched environment improves spatial learning and memory in MCAO rats.	An enriched environment increased numeric synaptic density in the parietal cortex and induced structural changes in synaptic junctions, decreasing the width of synaptic clefts and increasing the thickness of PSD in the hippocampus and parietal cortex. The enriched environment enhanced the expression of phosphorylated NMDAR1 and PSD-95.	Xu et al., 2009
pMCAO	The cage was a large wooden container (70 cm wide, 85 cm long, and 30 cm tall) with various toys, such as little houses, ladders, tubes, and moving wheels. Every 3 days, these toys were replaced with new forms and colors.	For 21 consecutive days	An enriched environment improves synaptic remodeling and motor function recovery in mice’s ischemic penumbra region after stroke.	Analysis of correlations revealed that the increased expression of GAP-43 and SYN was closely associated with the recovery of limb function in mice with ischemic injury.	Shi et al., 2023
pMCAO	The enriched environment group mice lived in 65 cm wide, 75 cm long, and 25 cm high cages with climbing ladders, exercise wheels, plastic tubes, toys, tunnels, running wheels, sheds, and decorations. To preserve environmental freshness, the circumstances above were changed every 3 day.	For 14 consecutive days	An enriched environment improves locomotion, balance and coordination, and spatial learning memory functions in post-stroke mice.	An enriched environment increases fibronectin type III domain-containing protein 5 (FDNC5) and BDNF expression in the ipsilateral cerebral cortex of mice after stroke.	Yu et al., 2020
tMCAO	The enriched environment cage (86 cm × 76 cm × 24 cm) has running wheels, climbing ladders, nest boxes, hammocks, tunnels, colored balls, and blocks. The lid holds water and a food container like the basic cage. For airflow, the lid has 3 cm openings.	Starting after 5 days of tMCAO and lasting for 21 days	Delayed exposure of cerebral ischemic rats to an enriched environment promotes the survival and density of newborn neurons in the hippocampus and the synaptic density of mature neurons, ultimately improving neurological function and spatial learning deficits.	Histone deacetylase 2 (HDAC2), synaptic-associated proteins, and brain-derived neurotrophic factor (BDNF) may be potential mechanisms for the positive effects of the enriched environment.	Tang et al., 2019
tMCAO	The enriched environment was constructed in a large wire cage (83 × 64 × 56 cm) with several objects (wooden ladders, running wheel, shelter, wooden bridge, wooden and plastic toys, aromatic cotton balls, and hanging chains). Rats reached for food and water through wooden ladders. Eight rats were housed per cage with gentle music and illumination alternating with dark every 2 s. To preserve novelty, these things were rearranged and lowered daily.	For 7 consecutive days	An enriched environment promotes neurogenesis in the subventricular zone after ischemic stroke.	Growth arrest and DNA-damage-inducible protein 45 β (Gadd45b) is associated with subventricular zone (SVZ) neurogenesis after ischemic stroke, and Gadd45b mediates enriched environment-induced SVZ neurogenesis via BDNF.	Tan et al., 2021
tMCAO	A huge plexiglass cage featuring a running wheel, catwalk, play items, and hidden tunnels to facilitate socialization, motor skill development, and other forms of multimodal training.	For 28 consecutive days	An enriched environment improves functional recovery and neurogenesis in aged stroke rats.	The enriched environment promotes neurogenesis by reducing post-stroke inflammation.	Gresita et al., 2022
tMCAO	Task-specific motor rehabilitation is performed by having post-stroke rats grasp sucrose pellets outside their cages for training. The rats are also housed in an enriched environment cage with various toys and novelty items that are changed regularly to promote recovery of synergistic function.	For 14 consecutive days	A rehabilitation program that combines task-specific motor rehabilitation training with an enriched environment can facilitate neurogenesis and functional recovery after a stroke.	Task-specific motor rehabilitation combined with enriched enrichment promotes neural regeneration and maturation of new neuronal dendrites by reversing histone deacetylase 6 (HDAC6) dysfunction caused by cerebral ischemia.	Sheu et al., 2019
tMCAO	Twenty-four hours after MCAO, rats were placed in large cages (50 × 75 × 90 cm) with novel things such as climbing ladders, plastic tunnels, shelters, toys, tubes of varying sizes, and running wheels for free-running.	Starting after 2 days of tMCAO and lasting for 18 days	The enriched environment promoted functional recovery, reduced infarct size, and enhanced angiogenesis.	The enriched environment may promote post-stroke angiogenesis through astrocytic interleukin-17A (IL-17A).	Chen et al., 2023
tMCAO	Ten mice were kept in 100-centimeter-cubic enclosures outfitted with tunnels, hideaways, toys, and running wheels to encourage free-range activity.	For 10 consecutive weeks	An enriched environment promotes functional recovery and angiogenesis in post-stroke mice.	The enriched environment promotes angiogenesis through the PI3K/AKT signaling pathway by promoting astrocyte high-mobility group box-1 (HMGB1) and interleukin-6 (IL-6) expression.	Chen et al., 2017

pMCAO, permanent middle cerebral artery occlusion; tMCAO, transient middle cerebral artery occlusion.

## Clinical research on enriched environment

Studies on enriched environments seem to focus on animal experiments, but our ultimate goal is to improve stroke patients’ prognosis and functional recovery in the clinical setting. In 2012, the first research team attempted to transfer this therapeutic strategy from animals to humans. This is undoubtedly exciting. The study included various recreational facilities in the enriched environment, including computers with Internet access, puzzles, board games, a coffee table, fiction and non-fiction books, and a dining area. In addition, patients were provided with Nintendo game consoles twice a week. Furthermore, this area is communal to allow patients to receive more socialization. Since each patient has different interests, patients are also allowed to choose the entertainment that interests them, such as music, number puzzles, etc. Patient’s families are also encouraged to participate. This article provides detailed and informative experimental designs for conducting studies in enriched settings in the clinical setting, such as how the enriched setting is designed, the inclusion conditions for subjects, the collection and analysis of data, and what data are collected. We can all find enlightening clues in this article (Janssen et al., 2012).

Previous research has demonstrated the positive effects of music on inducing voluntary movement, as music can distract people from their bodies while they are moving, thereby reducing their perception of movement and ultimately making it easier to complete (Dyrlund and Wininger, 2008). In addition, it has been found that auditory-motor Coupling, i.e., synchronization of body movements with the rhythm of music, can help individuals establish and strengthen social connections (Roerdink et al., 2009). Other researchers have found music beneficial for cognitive and motor function recovery in post-stroke patients (Särkämö et al., 2008; Särkämö and Soto, 2012). Combining music and therapy provides a more prosperous recovery environment for post-stroke patients than traditional rehabilitation methods. With this in mind, Preeti Raghavan and his colleagues have designed a treatment for post-stroke patients called Music Upper Limb Therapy-Integrated (MULT-I) (Palumbo et al., 2022). MULT-I is the combination of music therapy and occupational therapy. Patients receive MULT-I twice a week, and the whole treatment process lasts for 6 weeks. After 6 weeks, the Fugl-Meyer Scale results showed that the patient’s upper limb motor function had improved, and the patient’s sense of wellbeing and participation in the process was also higher. In addition, the study also conducted a 1-year follow-up to determine the effect of MULT-I on psychological and social aspects, and the results were also positive. Patients could choose their favorite music according to their preferences, and group therapy also enhanced the social interaction between individuals. Patients reported that MULT-I made the treatment process easier and more enjoyable.

## Future directions

In summary, an enriched environment can promote plastic changes and recovery in the brain after a stroke and reduce related symptoms. These findings provide new ideas for clinical treatment; for example, rehabilitation can be combined with enriched environments to accelerate patient recovery. However, further studies are needed to confirm the exact role of enriched environments on stroke recovery and the best way to apply them. In recent years, research on enriched environments has gradually expanded to the individual level because each person or animal experiences things differently in enriched environments. The brain is characterized by plasticity, so each of us has a different brain structure because of our experiences. The difference in brain structure affects our behavior, and behavior shapes our brain again, which forms a closed loop. This also coincides with the personalized medicine advocated in recent years. But many people have also raised questions about the enriched environment as a treatment method. The control group is usually rats in a standard environment when we study enriched environments. The standard environment is a shoebox-sized plastic cage with only water, food, and bedding (Coleman and Schapiro, 2021). Housing space for rodents is usually relatively limited due to laboratory constraints and cost considerations. This can result in animals being unable to engage in activities, explore and exhibit natural behaviors fully. Rodent housing in standard environments often lacks the richness and variety of environmental stimuli (Bradshaw and Poling, 1991; Van de Weerd et al., 1997). The lack of adequate opportunities for exploration and play may lead to behavioral degradation and boredom. Standard environments often do not fully simulate the conditions in which they live in their natural environment. For example, for cave-builder rodents, adequate underground exploration space cannot be provided. Studies have found that rats living in such standard environments develop behavioral abnormalities, become poor sleepers, and become more pessimistic (Burman et al., 2008; Brydges et al., 2011; Richter et al., 2012; Resasco et al., 2021). A meta-analysis showed that the traditional standard environment exacerbates the severity of diseases such as cancer, cardiovascular disease, stroke, anxiety, and depression. In addition, it increases mortality, and this negative effect is independent of species and gender (Cait et al., 2022). Thus, the standard environment appears to be a deprivation treatment rather than a baseline value. Conversely, the enriched environment corresponds to our normal human life, which seems to be one reason why animal experiments cannot be successfully replicated in the clinic. It is reassuring, however, that no evidence leaving the standard environment increases the variability of the data. Therefore, enriching the environment does not reduce the statistical power of the data (Wolfer et al., 2004; André et al., 2018).

As a therapeutic tool, enriched enrichment can offer many potential benefits. However, it is worth noting that it also has limitations that cannot be ignored. First, “enriched enrichment” is a relatively vague concept with no clear definition or consistent criteria. Different people and organizations may have different understandings of “enriched environments,” leading to confusion about creating and evaluating them. Second, although some research supports the positive impact of enriched environments on individual health and development, there is insufficient scientific evidence to demonstrate the effectiveness of enriched environments as a specific therapeutic tool. As a result, controversy remains regarding the therapeutic effects and mechanisms of enriched environments. Furthermore, enriched environment design is often based on the assumption that diversity and stimulation benefit all individuals. However, individuals may have different needs and responses, and some may be overly sensitive to overstimulation or environmental changes. Therefore, whether enriched environments are appropriate for all individuals requires more in-depth research and individualized consideration. Finally, enriched environment therapy requires long-term maintenance and management; otherwise, the recovery effect may gradually diminish. This may require long-term hospitalization or rehabilitation programs, increasing the treatment cost and burden.

## Author contributions

YH and Z-KG devised the whole structure of the manuscript. X-YS retrieved literature and did some analysis. P-PH wrote the manuscript. XB polished the manuscript. All authors contributed to the article and approved the final manuscript.
